# Patterns of internationalization and criteria for research assessment in the social sciences and humanities

**DOI:** 10.1007/s11192-016-1845-1

**Published:** 2016-02-02

**Authors:** Gunnar Sivertsen

**Affiliations:** Nordic Institute for Studies in Innovation, Research and Education (NIFU), P.O. Box 2815, 0608 Tøyen, Oslo, Norway

**Keywords:** Research assessment, Evaluation criteria, Social sciences, Humanities, Publication patterns, Internationalization, Societal relevance, Journals, Book publishing, Language, Web of Science, Scopus

## Abstract

This article investigates the developments during the last decades in the use of languages, publication types and publication channels in the social sciences and humanities (SSH). The purpose is to develop an understanding of the processes of internationalization and to apply this understanding in a critical examination of two often used general criteria in research evaluations in the SSH. One of them is that the coverage of a publication in Scopus or Web of Science is seen in itself as an expression of research quality and of internationalization. The other is that a specific international language, English, and a specific type of publication, journal articles, are perceived as supreme in a general hierarchy of languages and publication types. Simple distinctions based on these criteria are contrary to the heterogeneous publication patterns needed in the SSH to organize their research adequately, present their results properly, reach their audiences efficiently, and thereby fulfil their missions. Research quality, internationalization, and societal relevance can be promoted in research assessment in the SSH without categorical hierarchies of publications. I will demonstrate this by using data from scholarly publishing in the SSH that go beyond the coverage in the commercial data sources in order to give a more comprehensive representation of scholarly publishing in the SSH.

## Introduction

The presence of publications in Scopus or Web of Science (WoS) has increasingly become a criterion in evaluations of research in the social sciences and humanities (SSH). Some countries have even installed protocols for research evaluation or performance-based funding models where publications that are indexed by the commercial databases are treated separately in indicators of “internationalization” and “research quality”. In other countries, there is a general belief that research quality can be promoted in the SSH by expecting more publications in the limited number of international journals that have been selected for indexing. Consequently, for several years already, Elsevier and Thomson Reuters have experienced a pressure from researchers in the SSH to have more journals indexed. Both providers have responded by increasing the coverage of journals and book series, and, recently, even of books in the SSH. However, the coverage of the scholarly publication output in the SSH is still limited (Sivertsen 2014). The shortage is mainly due to the more heterogeneous scholarly publication patterns in the SSH where publishing in international journals is supplemented by book publishing and the use of journals in the native languages (Hicks 2004; Archambault et al. 2006; Engels et al. 2012; Sivertsen 2014).

Just as with the abuse of Journal Impact Factors in research assessment of individual performance in science, technology and medicine (STM), the ‘coverage criterion’ in the SSH represents an artefact which is external to and beyond the control of the scholarly norms and standards that it is sought to represent. It creates unnecessary tensions between fields in the SSH with different degrees of coverage in the databases. It also creates debates about what will happen to the use of books and native languages in the SSH. In these debates, the general development towards publishing in journals covered by Scopus or Web of Science is often perceived as “inevitable” and driven by new evaluation regimes, not by internal scholarly standards. In this study, I will develop an understanding of the processes of internationalization in the SSH which is independent of the ‘coverage criterion’ and instead related to concepts of field-specific research excellence and societal relevance in the SSH.

In a historical perspective, it is easy to demonstrate that the SSH are not originally “national” in their publishing practices. They started by being international within an academic elite. Latin was the first of several international languages that have been used during several centuries. The “nationalization” of the SSH is closely connected to the democratization of education and cultural and social life in the 20th century. Today, the quality and relevance of research in the SSH is not only checked by peers, but also directly by society. Internationalization is important for research quality and for specialization on new themes. However, the SSH would lose their *raison d’être* by disconnecting from the surrounding culture and society and by mainly communicating in international journals that are only read by peers abroad.

Research evaluation works in the space between observations and expectations when judging research performance. My contribution here will be to lay the ground for a renewed discussion of assessment criteria (representing expectations) in the SSH by using bibliometric methods and data (representing observations) to demonstrate the actual patterns and developments in scholarly publication practices from the perspective of internationalization.

## Methods

For the purpose of this study, data are needed that give a complete representation of scholarly publishing it the SSH, also of publications in books, series and journals not covered by Scopus or Web of Science. In 2005, Norway was the first country to establish a national information system with complete quality-assured bibliographic data covering all peer-reviewed scholarly publishing in the total higher education sector. The driver behind the creation of the system has been the so-called “Norwegian model”, which requires the bibliographic data for a publication indicators that serves a performance-based funding fomula (Schneider 2009; Sivertsen 2010; Ahlgren et al. 2012; Aagaard et al. 2015). The information system itself, which is now called CRISTIN (Current Research Information System in Norway) and has been expanded beyond the higher education sector, provides the main source of data for this study.

As we rely on data from one country only, the basis for generalizations can be questioned. In an earlier study (Ossenblok et al. 2012), we compared the publication patterns in the SSH in two countries, Flanders (Belgium) and Norway, with the use of data from similarly structured and defined comprehensive national systems. We could confirm the observation in an earlier study (van Leeuwen 2006) that *publication patterns differ between the disciplines of the SSH while they are similar across countries within the disciplines*. Even in disciplines with a nationally oriented publication pattern, the pattern itself is international. As an example, the publication pattern in sociology (degree of international publishing; percentage book publishing versus journal publishing; coverage of publications in the WoS) was much the same in the two countries and it also differed from that of economics in a similar way. In the present study, we assume that the disciplines of the SSH basically have specific publication patterns that are similar across countries.

The methodology of the bibliographic data collection in the Norwegian CRISTIN database (www.cristin.no) has been published earlier (Sivertsen 2010, 2014; Sivertsen and Larsen 2012). Scientific and scholarly publications of all fields are covered completely according to an agreed definition. Among other criteria, the definition demands originality and scholarly format in the publication and peer-review in its publication channels. All publication channels (journals, series, book publishers) and publication types (see below) are standardized in the database.

*Humanities* is defined in our study as the disciplines included in this major area in the OECD Field Classification.^1^ The *Social Sciences* are defined in the same way with the exception of Psychology, which we have not included in this study. Note that Law and Educational Research are classified as social sciences by OECD. In the following, we will perform the analysis both on the level of the two major areas and on the level of disciplines. We have selected History and Linguistics within the humanities, and Economics and Sociology within the social sciences, as cases for the analysis of disciplines.

Three supplementing data sets (A, B, C) will be used, each of them for a more specific purpose:

- For the analysis of publication patterns in the SSH down to the level of *individual researchers*, we use data from the above-mentioned CRISTIN system which is available only from 4 years 2010–2013. The unit of analysis is publications per researcher within a variable of three publication types (articles in journals or series with ISSN; articles in books; books) and a dichotomous variable of languages [Norwegian (the native language); International languages]. Only active researchers with at least two publications in the period are included. This criterion selects 1895 unique researchers in the humanities with 7145 unique publications, and 3229 unique researchers in the social sciences with 11,817 unique publications. Their publications are classified (in the OECD scheme) by discipline and major area on the basis of *the author’sinstitutional affiliation*.
- For the analysis of the *development* of publication patterns in the SSH over time, we use data that are defined and collected in the same way as in data set A, but aggregated at the level of disciplines. The data cover the years 2005–2011. The unit of analysis is publication per discipline (and major area) with the same variables of publication types and languages as in data set A. Data set B includes 14,558 unique publications in the humanities and 19,450 unique publications in the social sciences. Differently from data set A, these publications are classified (in the OECD scheme) by discipline and major area on the basis of *a journal classification* for all journal articles and a classification of *individual book titles* for books and articles in books. The latter quite laborious procedure has not been possible to continue after 2011.
- For the more specific analysis of the development of internationalization in the SSH over time, we use data from the *National Citation Report* 1981–2011 for Norway, a Thomson Reuters product that provides Web of Science data for bibliometric analysis at the national level. We combine the data source with another Thomson Reuters product, the *Journal Performance Indicators*, covering the same years. The unit of analysis is publications per journal covered by WoS in a given year. The field classification of journals in WoS has been mapped over to our use of the OECD classification. Publications are not double-counted if they are assigned to more than one category in WoS. The data include 2726 unique publications in the humanities and 8105 unique publications in the social sciences.

Note that the Norwegian data and the WoS data are comparable with regard to the type of publications that are usually included in bibliometric analysis. Only peer-reviewed publications representing original research are included in the Norwegian data. This limitation corresponds to the usual selection of ‘articles’ and ‘reviews’ for analysis in WoS data.

### Results, part I: characteristics of the publication patterns in the SSH: publication types

As seen in Table 1, publications in journals and series represent a little more than half of the publications in the humanities and two-thirds of the publications in the social sciences, indicating that book publishing is important as well, especially in the form of articles in books (edited volumes). There are, however, just as wide differences *within* each of the two major areas: Only 45 % of the publications in History are in journals, compared to 61 % in Linguistics. In Sociology, only 46 % of the publications are in journals, compared to 75 % in Economics.

**Table 1:** Number and percentage publications per publication type

	Humanities (*N*)	Humanities (%)	Soc sci (*N*)	Soc sci (%)
Books	328	4.6	273	2.3
Articles in books	2861	40.0	3640	30.8
Articles in journals or series	3956	55.4	7904	66.9
Total	7145	100.0	11,817	100.0

Based on data set A

The scholarly publication types in the SSH are often discussed as if they represent alternatives to each other: Is the use of one of the publication types increasing at the cost of the others? Are monographs becoming obsolete in the SSH? Before we study the trends, we shall observe an indication that the publication types are *supplementing each other rather than competing with each other*. As seen in Table 2, the numbers and percentages of *the researchers* that actually use a certain publication type are significantly higher than in Table 1, indicating that more than one publication type is often present in the publishing profile of an individual researcher. As an example, although less than a third of the publications in the social sciences are articles in books, more than half of the researchers are using this publication type.

**Table 2:** Number and percentage of the researchers using a publication type within 4 years

	Humanities (*N*)	Humanities (%)	Soc sci (*N*)	Soc sci (%)
Books	297	15.7	273	8.5
Articles in books	1187	62.6	1676	51.9
Articles in journals or series	1537	81.1	2775	85.9
Total (unique researchers)	1895		3229	

Based on data set A

Table 3 demonstrates to what degree the publishing profiles of individual researchers include more than one publication type. Even in the social sciences, where journal articles represent two-thirds of the output, almost half of the researchers who publish these articles also use other publication types. There are, however, differences at the level of disciplines. In Economics, only 29 % of the researchers who publish journal articles are also active in other publication types. The corresponding percentage in Sociology is 67 %. In the humanities, publishing in more than one publication type is the normal situation in all disciplines.

**Table 3:** Number and percentage of the researchers using a specific publication type that also uses another publication type within 4 years

	Humanities (*N*)	Humanities (%)	Soc sci (*N*)	Soc sci (%)
Books	265	89.2	250	91.6
Articles in books	891	75.1	1275	76.1
Articles in journals or series	930	60.5	1291	46.5

The percentages are related to the numbers (*N*) in Table 2. Based on data set A

So far, we can conclude that book publishing and journal publishing seem to supplement each other rather than represent alternatives in the SSH. We will return to a possible explanation for this in the discussion at the end.

### Results, part II: characteristics of the publication patterns in the SSH: language

We now turn to another dimension in the publication patterns of the SSH—the language dimension. In non-English speaking countries, the use of the native language in scholarly publications is an indication that the publication is mainly oriented at a national or regional audience of readers in which not only peers, but also students, teachers, professionals, journalists, policy makers and a wider public may be reached as well. Since scholarly publications in the native languages are relatively frequent in the SSH, publishing in an international language is, on the other hand, not the normal situation, as in the sciences, but a clear expression of an ambition to reach an international audience of experts in the field. In Norway, the international language in the social sciences is almost exclusively English. The same is true for most of the disciplines in the humanities, but here, German and French are also considered international languages. In philological disciplines, the language of the object of study also functions as an international language, e.g. Russian in Slavonic studies or Portuguese in Romance studies. For this reason, we use the more general term “International languages” as opposed to the native language in our data, Norwegian.

Table 4 shows that in both the humanities and the social sciences, the majority of scholarly publications are in the international languages. However, publications in the native language are much more frequent than in the sciences (for an empirical comparison based on similar data, see Sivertsen and Larsen 2012), indicating that such publications have a specific role in the SSH. As with the publication types, there are wide disciplinary differences *within* the two main areas of research: Publications in the international languages represent 85 % of the scholarly output in Economics and 83 % in Linguistics, but only 70 % in Sociology and 56 % in History.

**Table 4:** Number and percentage publications per language type

	Humanities (*N*)	Humanities (%)	Soc sci (*N*)	Soc sci (%)
International language	4368	61.1	8666	71.7
Norwegian language	2777	38.9	3418	28.3
Total	7145	100.0	11,817	100.0

Based on data set A

Again, the question may be raised: Are the native and international languages *supplementing each other, or are they competing as alternatives*? By going down to the level of individual researchers, we can observe in Table 5 that high proportions of the researchers combine both types of languages in their publication practice. While a majority of researchers publish in the international languages, there is *no minority of researchers* publishing in the native language only. Researchers in the SSH are *normally bilingual* in their publication practice (if their native language is not English).

**Table 5:** Number and percentage of the researchers using international and native languages in their scholarly publications within 4 years

	Humanities (*N*)	Humanities (%)	Soc sci (*N*)	Soc sci (%)
International language	1482	78.2	2687	83.2
Norwegian language	1228	64.8	1725	53.4
Total (unique researchers)	1895		3229	

Based on data set A

A more general conclusion from the results so far, is that although the *majority of publications* in the SSH are published in journals and in international languages, *the majority of researchers* are publishing in books and in the native language as well. Is this picture changing?

### Results, part III: developments in the publication patterns in the SSH

**Fig. 1:**
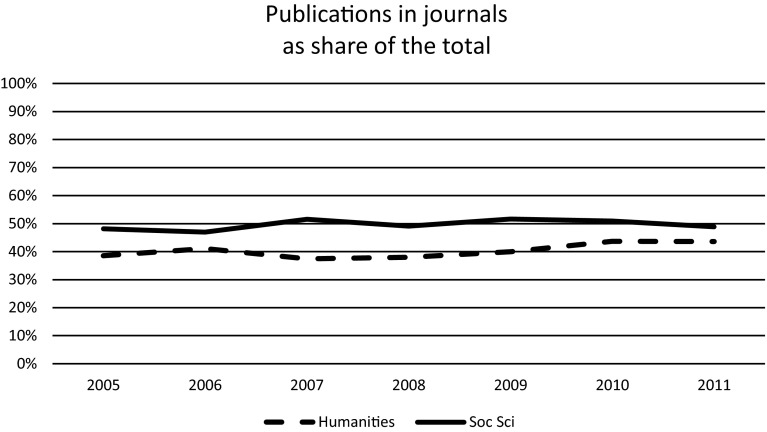
Scholarly publications in journals as a percentage of the total, which also includes articles in books and books. Based on data set B

**Fig. 2:**
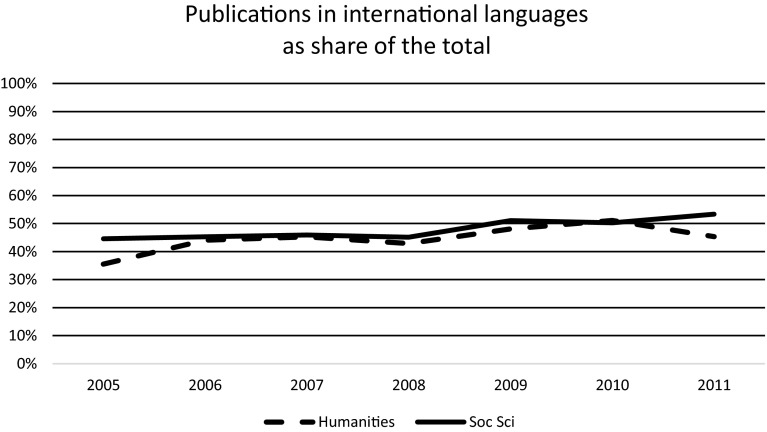
Scholarly publications in international languages as a percentage of the total, which also includes publications in the native language. Based on data set B

To study the developments, we use data set B, by which it is possible to cover a longer period of time. A limitation, however, is that we can only study publications at the level of disciplines, not at the level of individual researchers. Another difference is that data set B includes all scholarly publications from Norway’s higher education sector while only the more active researchers (with at least two publications in the period) are represented in data set A. This difference explains why the share of publications in journals and in international languages are slightly lower in Figs. 1 and 2 below than they are in Tables 1 and 4 above.

The general picture, however, is that the publication patterns in the SSH are quite stable, both with regard to publication types (Fig. 1) and the use of international versus native languages (Fig. 2). In relative shares, the uses of international languages and of journals are increasing, but not by a high rate. In absolute numbers, there is no in reduction book publishing or in the use of the native language, since in data set B, which we are using here, there was an increase in the total number of publications by more than 50 % between 2005 and 2011.

We return to data set A with data on individual researchers, including their age. Here, it is possible to detect possible changes in the publication patterns in a different way. By constructing two cohorts according to age—researchers younger than 45 years and researchers older than 55 years—we can measure the tendency to publish in journals and in international languages as percentages of all publications in each cohort. The results are shown in Table 6. There is a clear indication of a generation shift in the publication practices, but the shares and the differences are still not high enough to conclude that one type of languages or publications is taking over at the cost of the other types.

**Table 6:** Percentage publications in journals (vs. books) and in international language (vs. native language) in two cohorts of researchers, one with members younger than 45 years, and one with members older than 55 years

	Humanities younger (%)	Humanities older (%)	Soc sci younger	Soc sci older (%)
Publications in journals	64.5	52.2	72.4	63.1
Publications in international languages	64.5	56.3	75.4	69.1

Based on data set A

Instead, our conclusion so far is that the normal publication practice in the SSH, in which both types of languages, and books as well as journals, are used for scholarly publishing by the majority of researchers, seems to prevail during a period of internationalization. We will discuss the possible reasons for and consequences of this observation at the end. First, we will try to describe the process of internationalization more closely.

### Results, part IV: patterns of internationalization in the SSH

Patterns of internationalization can be studied in bibliometric data in several ways. One of the most used methods is to study co-authorship relations in articles with addresses in more than one country (Luukkonen et al. 1992). This method is known to have limitations in the SSH because single-authored articles are dominating in these areas by tradition. We do observe, however, that multi-authorship and international collaboration (based on the authors’ addresses) are increasing even in these areas. These observations are based on our data set C of Norwegian WoS-articles:

In the 1980’ies, there was on average 1.2 authors per article in the humanities, and only around 5 % of the articles had addresses in other countries than Norway. In the latest years, the average number of authors has increased to 1.5 and the percentage of articles with evidence of international collaboration is around 15 %. In the social sciences, there was an average of 1.3 authors per article in the 1980’ies. International collaboration was visible in around 12 % of the articles. These numbers have lately risen to 2.3 and 37 %. The SSH certainly follow the general pattern of *increasing multi*-*authorship and international collaboration in research*.

**Fig. 3:**
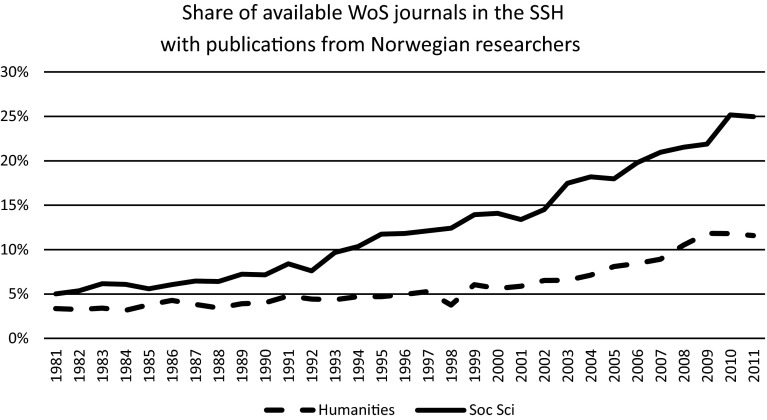
Percentages of available WoS journals in the social sciences and humanities that published articles from Norway 1981–2011. Based on data set C

Data set C can also be used to expose another dimension in the internationalization of the publication patterns of the SSH, namely *specialization*. We will do a stepwise analysis to show this. Firstly, in Fig. 3, we can note that WoS articles from Norway in the SSH are published in an increasing share of the available journals that are indexed by WoS.

The increases shown in Fig. 3 exceed the general expansion of articles and journals in the SSH in WoS in the same period. They also exceed the increase in the number of SSH articles in WoS from Norway. Consequently, we observe in Fig. 3 a *deconcentration* of the articles in a higher number of journals. With a closer look, we also observe that the trend is a move from a core of close-at-hand disciplinary journals to fully international journals that represent *specialties within disciplines* or a *cross*-*disciplinary thematic scope*. We can use the same four disciplines as before for the examples:

- In History, most WoS publications in English from Norway in the 1980’ies were published in *Scandinavian Journal of History*. Today, this journal has lost its central role (down from 57 % of the articles in 1981–1985 to 16 % of the articles in 2010–2014). Publishing has increased in international and more specialized journals such as *Historical Social Research*, *International Journal of African Historical Studies*, and *Cold War History*.
- In Linguistics, no Scandinavian journal was indexed an early stage, but general disciplinary journals such as *Lingua* had the central role (down from 15 to 7 %). Lately, there are relatively more Norwegian publications in more specialized journals such as *Journal of Pragmatics, Journal of Neurolinguistics,* and *International Journal of Bilingualism*.
- In Economics, *Scandinavian Journal of Economics* used to publish a very high share of the Norwegian WoS articles in its discipline (down from 19 to 2 %). Now, more specialized international journals have taken on just as an important role. Examples: *Environmental & Resource Economics*, *Health Economics*, and *International Tax and Public Finance*.
- In Sociology, *Acta Sociologica*, the journal of the Nordic Sociological Association, was the main publication channel addressing an international audience in the 1980’ies (down from 38 to 6 %). Today, more specialized or interdisciplinary journals such as *Social Indicators Research*, *Media Culture & Society*, and *European Societies*, play a more important role.

It is probably not only from the perspective of one country that the process of internationalization in the SSH can be detected also as a process of specialization reflected in a deconsentration of the publishing pattern. A closer study of the journal market itself, or of the new journals that have been added to WoS or Scopus from year to year, would probably reveal the same trend. It tells us something about what research in the SSH might gain from internationalization: Communication between experts in areas where there are low numbers of active researchers in each country.

Returning to data set B, which gives a more complete picture of the publishing patterns than the WoS-based data set C does, we see at clear pattern where the *national* journals take on the general and disciplinary role as scholarly and professional meeting places for original research of particular national interest along with debates, book reviews and information. In Norway, there is only one or two such journals in each discipline (in the four disciplines we use as examples, the national journals are named *Historisk tidsskrift*, *Norsk lingvistisk tidsskrift*, *Samfunnsøkonomen* and *Sosiologisk tidsskrift*). These few national journals represent, however, between one-third and a half of the total output of scholarly journal articles in their disciplines. Hence, the deconcentration and specialization on the international level is matched by a concentration of articles in more general disciplinary journals on the national level. Again, the different roles of the national and international journals in the SSH indicate that they *do not represent competing alternatives* in the publication pattern, but rather that *they supplement each other*.

## Discussion and conclusions

To sum up, we have seen that:

- Publishing in books *and in* journals is the normal practice for the majority of researchers in the SSH
- Publishing in the native language *and* in international languages is the normal practice for the majority of researchers in the SSH
- There is a process of internationalization in the SSH which also reflects a process of specialization. Still, the process does not indicate a turn to a more one-dimensional publication pattern with the use of only one language and format.

The stability of the publication patterns and their differences within the SSH indicate that the choice of language and publication type is not just a question of new trends versus old traditions. Publication patterns are more deeply rooted in scholarly norms, methods and practices. The monograph, the edited book and the journal article represent different methodologies that may all need to be used at different times. The choice of language depends on the international scholarly relevance of the research versus the societal relevance for the culture and society being studied. One and the same research project may well contribute with different parts to both dimensions. As mentioned in the introduction, the SSH would lose their *raison d’être* by disconnecting from the surrounding culture and society and mainly communicating in international journals that are only read by peers abroad. At the same time, publishing in those specialized journals on the international level is necessary in order to be confronted with and inspired by the scholarly standards, critical discussions and new developments among other experts in the field.

In the context of criteria for research evaluation in the SSH, there is a need to accept that none of the alternatives in the two dimensions of the scholarly publication patterns that have been described here—language and publication type—can be regarded as more valuable alternatives. All of them contribute—with different roles and connected to different methodologies, audiences and feedbacks—to research excellence and societal relevance in the SSH. This observation does not, of course, set aside any judgment of quality differences between scholarly publications or publication channels within each category of languages or publication types. There is no reason, however, for applying a general hierarchy of languages or publication types in assessment of research in the humanities and social sciences, e.g. by rating all journal articles in the English language as superior to other publications.

Furthermore, the coverage in Scopus or the Web of Science of the scholarly publishing pattern in the SSH is far from complete (Sivertsen 2014). Even in the category of international journals used by Norwegian researchers (data set B), we can see that the coverage of articles is below 50 % and has been *decreasing* since 2005. (The expansion of Web of Science and Scopus in the SSH has not kept up with the rapid development of new international and specialized journals in these fields). For this reason, and because the coverage of publications in books and in the native languages is even more limited, *coverage in a commercial indexing service* should not be used as a criterion for research quality or an indicator of internationalization in the SSH. Neither should *journal impact factors* be used for a similar purpose. The trend towards internationalization in the SSH is reflected in an increased use of specialized journals, and not—as in the sciences—in increased publishing in a few core journals with high citation rates and a rapidly increasing volume of articles per year. Internationalization in the SSH should therefore be stimulated without introducing general hierarchies of languages and publications types or simple coverage criteria that overlook how different qualities of research are realized in these areas.

## Footnotes

1 OECD: revised field of science and technology (FOS) classification in the Frascati manual, version 26-Feb-2007, DSTI/EAS/STP/NESTI (2006)19/Final.
